# The hazards of chasing subgroups in neutral stroke trials

**DOI:** 10.1186/s42466-025-00369-0

**Published:** 2025-03-11

**Authors:** Philip M. Bath, George Howard, Werner Hacke

**Affiliations:** 1 Stroke Trials Unit, Mental Health & Clinical Neuroscience, Queens Medical Centre, University of Nottingham, Nottingham, NG7 2UH UK; 2 School of Public Health - Biostatistics, The University of Alabama at Birmingham, Birmingham, AL USA; 3 Department of Neurology, Ruprechts Karl University Heidelberg, Heidelberg, Germany

**Keywords:** Randomised controlled trial, Subgroup, Analysis, Neutral, Design

## Abstract

**Background:**

The majority of randomised controlled trials in acute stroke and many for prevention are neutral, i.e. they failed to reach statistical significance. However, many of these will find apparent benefit in a component of a subgroup, findings which may be ‘chased’ in a follow-up trial. The evidence to date is that these follow-on trials are very likely to be neutral.

**Findings:**

We discuss the issue of chasing subgroups in neutral trials and illustrate the challenges in five pairs of exemplar acute stroke trials. Problems in the exemplar trials include failing to define the subgroup in advance or even changing its definition, failing to show that both the interaction test and the primary outcome in the component were statistically significant, failing to publish additional information on the positive subgroup component, having too many subgroups, failing to make the follow-on trial large enough and failing to report the findings of the follow-on trial.

**Conclusion:**

When chasing a positive component in a subgroup, it is vital that the subgroup: should be plausible biologically, defined a priori and have a significant interaction test. Further the number of subgroups should be limited and the component of interest should be statistically significant. Explanations should be given as to why the component is positive and other components of the subgroup are negative. Other outcomes should also show potential benefit. Unless this guidance is followed, it is highly likely that follow-on trials will be neutral as has occurred previously.

## Background

Large phase III and IV randomised control trials in acute stroke typically study the effect of an intervention on a clinical functional primary outcome such as the modified Rankin Scale. [1] The effect of the intervention on the primary outcome in pre-specified subgroups of patients may then be assessed. Occasionally, the trial’s power calculation is based on a subgroup of interest rather than for the whole trial [2] and although this might be ideal [3], it is unusual because of the inflationary impact on sample size and the raising of potential ethical concerns for needlessly exposing patients to experimental treatments in an over-powered trial [4].

A subgroup is a characteristic measured at baseline which comprises two or more components, i.e. sex is a subgroup and its components comprise female and male participants. Subgroup analyses may be performed for a variety of reasons: first, to investigate the consistency of treatment effects across clinically important groups of participants in a statistically positive or negative trial; second, to investigate the treatment effect across different subgroups within an overall statistically neutral/non-significant trial; third, to assess safety within one or a few subgroup(s); and last, to establish efficacy in the subgroup of interest when included in a confirmatory assessment following a previous trial [5]. For the purpose of this review, a positive study is defined as “one in which the intervention has statistically significant beneficial results relative to the comparator”, a negative study is defined as “one in which a statistically significant harm was found” and a neutral study is defined as “one in which there was no statistically significant difference between the intervention and the comparator” [6–8].

Typically, the sample size is set for the whole trial, subgroup analyses should be considered purely for generating new hypotheses, i.e., what participant characteristics might modulate the effect of treatment on outcome. Prior to hypothesis testing, analyses should test whether there is a statistical interaction between the effect of treatment across the subgroup, i.e., whether there is evidence that effect of treatment on outcome differs across components of the target subgroup. If the interaction analysis is statistically significant, then the primary analysis can meaningfully be tested in each component of the subgroup. Conversely, if the interaction analysis is statistically non-significant, then further analyses of each component of the subgroup is questionable and introduces the possibility to interpret observed differences between the treatment effects in the subgroups as meaningful differences between the groups. A caveat here is that interaction testing is usually underpowered statistically. Interaction testing may be based on unadjusted, or covariate-adjusted analyses in the case where the primary analysis included adjustment for covariates, with covariates comprising prognostic factors such as age, sex, stroke severity and time from ictus to randomisation or treatment [9].

If overall efficacy is seen across the trial and this is present especially in one or more components of a subgroup, particularly if there is a qualitative interaction with effects in subgroup components going in opposite directions [10], then mechanistic hypotheses may be generated. For example, one reason the effectiveness (on an absolute scale) of carotid endarterectomy may decline with time following the index event is because the risk of recurrence similarly declines with time [11]. As a result, a further trial, if warranted, might wish to focus on the ‘early’ component of the time to randomisation subgroup.

The question then arises as to the value of subgroup interaction testing in neutral rather than positive or negative trials. Some methodologists argue that these should not be performed [12, 13], largely because any statistically significant subgroup interaction is likely to reflect chance. In contrast, since subgroup assessment is done to test hypotheses, an alternative view is that it is important to perform such analyses since it may make sense to test the result of a positive subgroup in a following trial in the hope that the second trial will demonstrate efficacy; such replication will usually control the likelihood of a spurious finding arising from the testing of multiple subgroups [13]. An additional argument is that the likelihood of a type 1 error for an assessment of interaction is largely independent of the main effect. Detailed guidance on this approach is given by regulators [14].

However, the potential benefit of testing interventions in a specific subgroup in acute stroke is low and multiple failed attempts have been published, some of which we describe here as exemplars. Indeed, we are not aware of any positive examples for individual trials in stroke. Whilst we would not want to recommend never testing positive subgroups derived from neutral trials, there are a number of rules that should be followed. The component of a subgroup should only be ‘chased’ in a further trial if: i) the components of the subgroup are defined before analysis (‘*pre hoc*’); ii) differences between them are biologically plausible; iii) the statistical interaction test is significant; iv) the primary outcome in the subgroup’s component of interest is statistically significant; v) there were not an excess number of subgroups being tested; and vi) there are plausible explanations for why the positive component of the subgroup responds and why the non-positive component(s) of the subgroup do not respond and may even suffer with treatment.

Using some of these rules, we can hypothesise a neutral trial of a novel intervention where participants treated earlier responded significantly whilst those treated later did not respond to treatment. Since existing treatments such as thrombolysis are known to be more successful if given early [15], it is plausible that other interventions might equally have a time dependent-effect. The subgroup of time was defined *pre hoc*, the interaction test is significant, the analysis in those patient treated early shows significant benefit and there are few other subgroups being tested. As a result, there is reasonable supporting evidence to further test the intervention in this positive subgroup component.

Here, we present several exemplar pairs of neutral completed trials where the first study had an apparent positive subgroup component and was followed by a second trial which only recruited participants with that characteristic. We report a narrative rather than a systematic review, partly because focussing on exemplars is more informative and partly because electronic searches for positive subgroup components does not adequately discriminate between relevant and non-relevant trials.

## Exemplars

### Completed trials

#### Piracetam for ischaemic stroke

Piracetam is a derivative of gamma-aminobutyric acid (GABA). GABA agonists/mimics enhance GABA_A_ receptor activity and so cause hyperpolarisation of neuronal membranes to prevent glutamate excitotoxic effects and calcium influx [16]. Both GABA agonists/mimics in general, and piracetam specifically, have been shown to be neuroprotective in multiple preclinical models of stroke [17, 18]. Unfortunately, a metaanalysis of preclinical studies of piracetam for preclinical stroke found few studies which were of low quality scoring just 4 (interquartile range 4–6) out of 10; potential publication bias was also present [18]. Hence, clinical studies are based on potentially weak preclinical data.

**Fig. 1:**
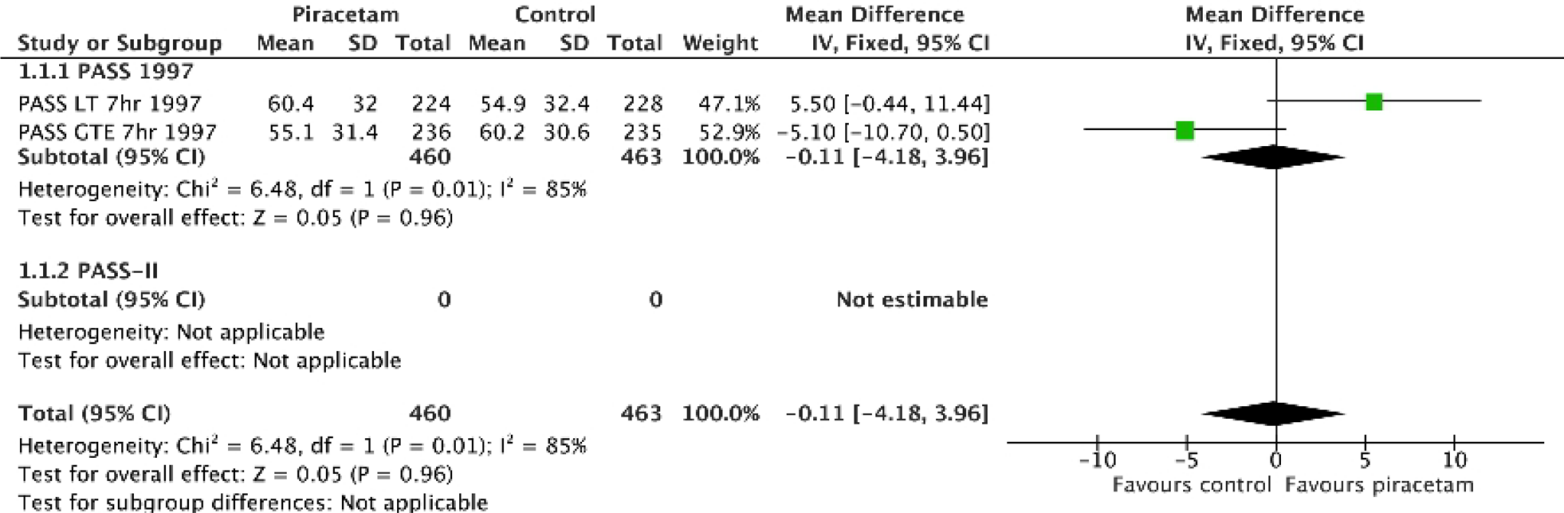
Effect of piracetam on the Orgogozo stroke severity/impairment scale (high scores indicate recovery) in acute ischaemic stroke. Data were extracted from the PASS publication; [19] the PASS >  = 7 h data were estimated from the publication. No data were available for PASS-II. The mean difference (fixed effect) was calculated in Cochrane REVMAN software; a mean difference < 0 implies a good outcome. A positive mean difference implies a good outcome. The overall effect of piracetam was neutral. The post hoc interaction test between early (< /LT 7 h) and later (> = /GTE) treatment was significant. The component of interest (randomisation within 7 h) was non-significant. The results of the PASS-II follow-up trial are unpublished

The Piracetam Acute Stroke Study (PASS) assessed the safety and efficacy of piracetam in patients with ischaemic stroke within 12 h of onset (Table 1) [19]. PASS set a *pre hoc* subgroup of testing the interaction by time to treatment with a cut point at six hours, i.e. treated within 6 h or between 6 and 12 h after stroke. The effect of piracetam on the primary outcome of Barthel Index was neutral, both overall and in those participants treated within six hours. However, a non-significant benefit was seen in those treated within seven hours, a post hoc cut point, and especially in those with moderate-to-severe stroke [19]. These results led onto the piracetam acute stroke study-II (PASS-II) trial [20]. However, this trial was stopped early for futility and has never been published [21]; further, [21] and the data were not made available by the manufacturer for meta-analysis on request (Table 1, Fig. 1) [22]. We can only assume that the data for PASS-II were neutral or even negative. Key concerns here are that: i) PASS-II was based on a post hoc subgroup in PASS; ii) no interaction test was reported for the analysis based on dividing the data at 7 h; iii) the subgroup component of interest of treatment within seven hours was not statistically significant; iv) no explanations were given for why the subgroup within 7 h was more plausible than within 6 h; and (v) why piracetam might be effective within 7 h but potentially hazardous when given later. Further, the results of the PASS-II trial have never been published and its data have not been made available for meta-analysis [22] amounting to publication bias.

**Table 1:** Summary information from exemplar completed trials. Point estimates (95% confidence intervals) are given for a poor outcome

Intervention	Piracetam	Clomethiazole	Surgery	Glyceryl trinitrate, GTN	Nerinetide, NA1
First trial	PASS [19]	CLASS [24]	STICH [32]	ENOS [41]	ESCAPE-NA1 [55]
Target patient group	AIS	AIS	ICH	AIS/ICH	AIS/LVO
Trial size, N	927	1304	1033	4011	1105
No. of subgroups	?	?	12	15	11
Positive subgroup	Time from onset to randomisation	Severe stroke impairment (SSS [94])	Depth of haematoma	Time from onset to randomisation	Alteplase
Subgroup defined *pre hoc*?	No	Yes	Yes (minimisation)	Yes	Yes (stratification)
No. of subgroup components	2	2	2	5	2
Positive component	Time < 7 h	SSS < ~ 28	Superficial haematoma	< 6 h [44]	No alteplase
Interaction p term	Not reported	*P = *0.030 (unadjusted?)	*P = *0.020 (unadjusted)	*P = *0.031 (unadjusted)	*P = *0.0330 (unadjusted?)
Interaction p value adjusted for multiple testing	Not reported	No?	No	No	No
Bonferroni-adjusted p-value cut-point	Not reported	?	*P < *0.0042	*P < *0.0021	*P < *0.0045
Published subgroup	No	CLASS-TACS [30]	No	ENOS-early [44]	Health economics [95]
Target patient group	Hyperacute AIS	Severe stroke syndrome TACI [29] (as surrogate for impairment)	Superficial ICH	Hyperacute AIS/ICH	No alteplase
Positive component size (%)	452/927 (48.8)	546/1360 (40.1)	517/959 (53.4)	271/4011 (6.8)	446/1105 (40.4)
Result in positive component	MD 5.5 (?, ?), *p = *0.07	OR 0.62 (0.43–0.88), *p = *0.008	OR 0.69 (0.47–1.01), *p = *0.051	cOR 0.51 (0.32–0.80), *p = *0.004	OR 0.85 (0.72–0.99), *p = *?
Benefit in secondary outcomes	Yes: BI 58.6 (42.8) vs 49.4 (43.2), *p = *0.02 No: death	Yes: impairment (SSS)	Yes: evacuation by craniotomy (*p = *0.07)	Yes: death, disability, mood, cognition, quality of life [45]	Yes: death, infarct volume
Supportive meta-analysis	ND	?	No	Yes [45, 96]	No
Second trial	PASS-2 unpublished [22]	CLASS-I [27]	STICH-2 [33]	RIGHT-2 [42]	ESCAPE-NEXT (https://www.medscape.com/viewarticle/997312?form=fpf)
Target patient group	Hyperacute AIS, < 7 h	Severe stroke syndrome TACI [29]	Superficial ICH	Ultra-acute AIS/ICH	AIS/LVO
Trial size, N	?	1198	601	1149	850
Primary result of second trial	Unpublished, presumably neutral, possibly negative	Neutral, OR 1.23 (0.95, 1.61), *p = *0.11	Neutral, OR 0.86 (0.62, 1.20), *p = *0.37	Neutral, OR 1.25 (95% CI 0.97, 1.60), *p = *0.083	Neutral, poor outcome (mRS > 2) nerinetide 54.6% vs placebo 54.3%
Supporting evidence	Piracetam is a nootrope	Clomethiazole is a GABA-mimetic and antagonises glutamate excitotoxicity. [16] Preclinical data positive for early treatment. [17]	Surgery reduces haematoma size, pressure and noxious chemicals and improves penumbral perfusion	Levels of NO, a key vasoregulator, are low in stroke. [43] Preclinical data positive for early treatment. [35] Pilot RIGHT trial showed efficacy. [40]	Nerinetide perturbs post-synaptic density protein 95 (PSD-95) inhibiting neuronal excitotoxicity
Weakness of subgroup testing	Post hoc, interaction term not given, subgroup non-significant	Post hoc subgroup, i.e. switch from severe impairment (SSS) to severe syndrome (TACS)	Too many subgroups? Subgroup non-significant. Only two components	Too many subgroups? Small sample size in positive component	Too many subgroups?

AIS: acute ischaemic stroke; BI: Barthel index; LVO: large vessel occlusion; MD: Mean difference; ND: not done; OR: odds ratio; SSS: Scandinavian stroke scale; TACS: total anterior circulation syndrome

#### Clomethiazole for ischaemic stroke

**Fig. 2:**
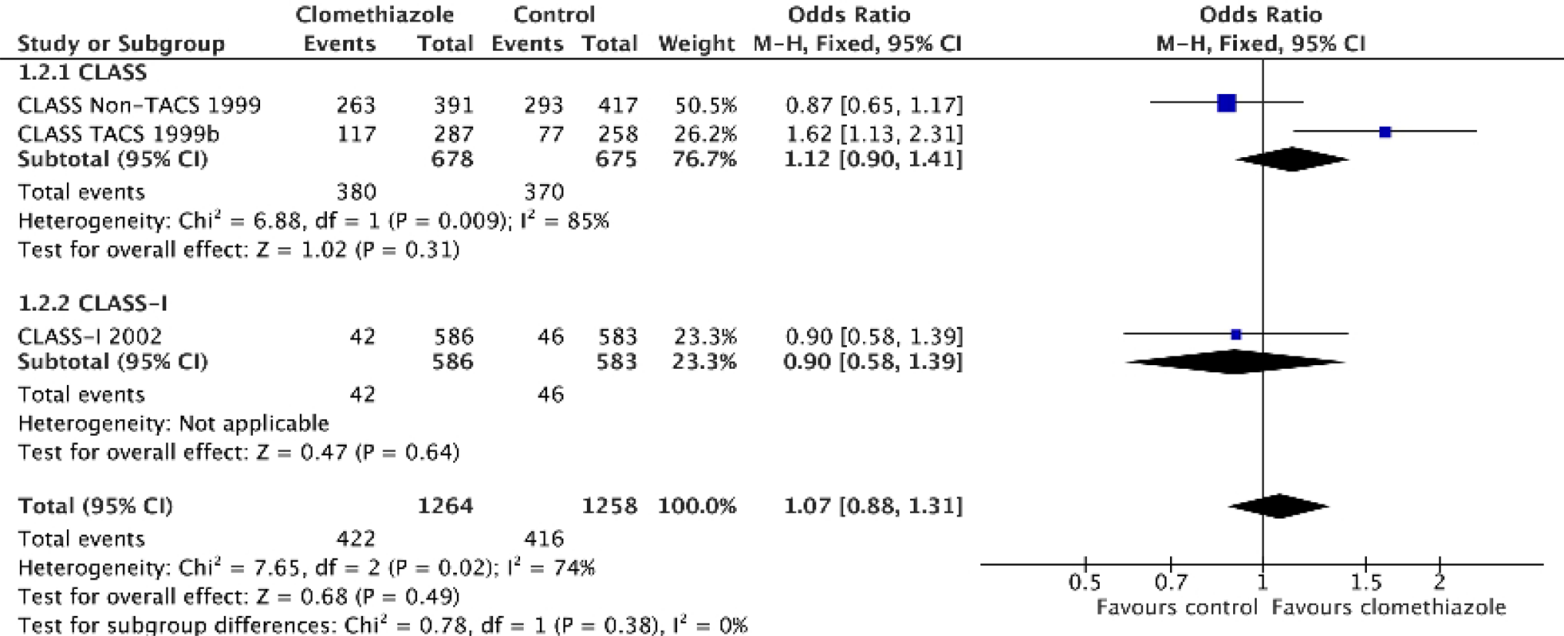
Effect of clomethiazole on Barthel index ≥ 60. Data were extracted from the CLASS and CLASS-I publications. [24, 27] The Mantel–Haenszel test in Cochrane REVMAN software was used with no adjustments. An odds ratio > 1 implies a good outcome. The overall effect of clomethiazole in CLASS was neutral. The post hoc interaction test between TACS and non-TACS was significant. The component of interest (TACS syndrome) was significant. The CLASS-I follow-up trial was neutral

Clomethiazole is another GABA-mimetic and has been tested in a limited number of preclinical stroke models [23]. Four clinical acute stroke trials have been reported [24–27]. Although the Clomethiazole Acute Stroke Study (CLASS, Table 1) was neutral in 1360 participants treated within 12 h of ischaemia onset [24], a statistical interaction between stroke severity (assessed using the Scandinavian stroke scale [SSS] [28] and chosen a priori) proved significant (*p = *0.030), and clomethiazole appeared to improve day 90 outcome in those patients with a severe stroke syndrome (odds ratio, OR 0.62, 95% CI 0.43–0.88, *p = *0.008). In a post hoc analysis, an interaction was found between stroke syndrome and treatment, specifically between those participants presenting with a total anterior circulation syndrome (TACS, the most severe clinical syndrome [29]) versus those without a TACS (*p = *0.038). Apparent efficacy in the TACS population was presented in the primary publication and described in more detail in a secondary publication [30]. These clinical observations along with preclinical data led onto the clomethiazole acute stroke study in ischemic stroke (CLASS-I) trial which focussed on patients presenting with a TACS syndrome within 12 h; [27] however, this second trial was neutral (Table 1, Fig. 2). The main weaknesses are: i) that it is not clear why GABA-mimetics would be beneficial in severe stroke but potentially hazardous in milder stroke, and ii) why a post hoc definition of severity (TACS) was used to define the inclusion criteria for the subsequent trial rather than the *pre hoc* SSS measure.

#### Surgery for intracerebral haemorrhage (ICH)

**Fig. 3:**
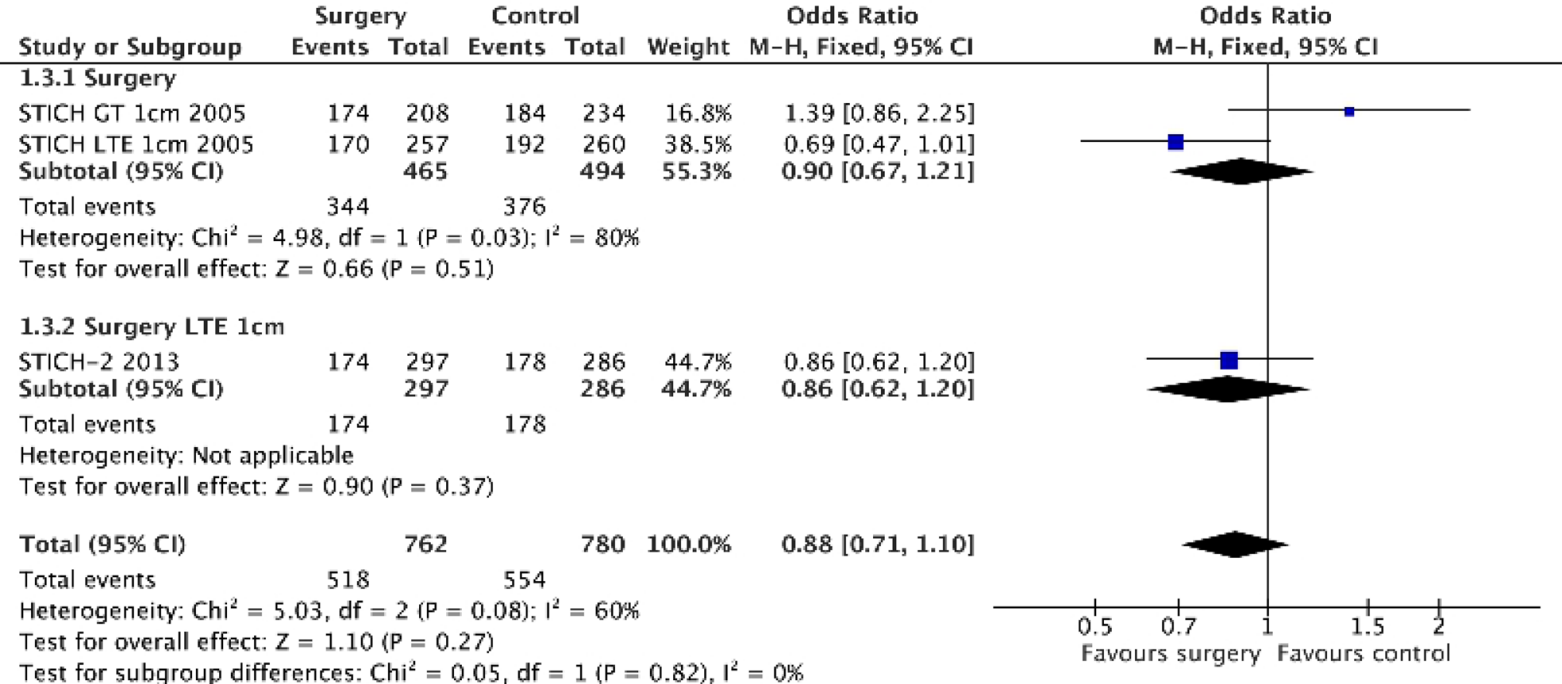
Effect of surgery for acute intracerebral haemorrhage on extended Glasgow Outcome Scale. Data were extracted from the STICH-1/2 publications. [32, 33] The Mantel–Haenszel test in Cochrane REVMAN software was used with no adjustments. An odds ratio < 1 implies a good outcome. The overall effect of surgery in STICH-1 was neutral. The post hoc interaction test between surgery for superficial and deeper haematoma is significant. The component of interest (superficial haematoma) was non-significant. The STICH-2 follow-up trial was neutral. Of note, meta-analysis of participants in STICH-1/2 with superficial haematoma is nominally significant, odds ration 0.78 (95% confidence intervals 0.61–1.00), *p = *0.05 with no heterogeneity

Numerous trials have assessed the effect of surgical management of intracerebral haemorrhage [31]. Surgery reduces haematoma size, pressure and noxious chemicals and probably improves penumbral perfusion [32]. In the largest trial, Surgical Trial in Intracerebral Haemorrhage (STICH), the effect on the primary outcome was neutral (Table 1); however, a significant interaction between depth of the haematoma (pre-specified as superficial ≤ 1 cm vs deeper > 1 cm from surface of cortex) and treatment (*p = *0.02) was detected [32]. Although the interaction term was significant, the effect of surgery in the superficial group did not quite reach statistical significance (OR 0.69, 95% CI 0.47–1.01, *p > *0.05). On the basis of these data, a second trial, STICH-II, recruited patients with superficial haematoma; it too was neutral result (Table 1, Fig. 3) [33]. The principle concern in chasing this subgroup is that the original analysis of efficacy in the subgroup of patients with superficial haematoma was not statistically significant. Further, the follow-on trial was smaller than the first trial which will have reduced its chance of being positive, as discussed below.

Of note, the recently published Early Minimally Invasive Removal of Intracerebral Haemorrhage (ENRICH) phase IIb trial of surgery [34] suggested that minimally invasive trans-sulcal parafascicular surgery might reduce poor functional outcome after ICH but with efficacy localised to lobar ICH and potential hazard present in basal ganglia presentation. These results are compatible with those seen in STICH-1. A follow-on trial is planned in patients with lobar ICH.

#### Glyceryl trinitrate (GTN) for lowering blood pressure

GTN (nitroglycerin) is a nitric oxide (NO) donor. Both NO donors in general and GTN specifically have been tested in preclinical models of stroke [35], including in large mammals (sheep) [36]. Whilst NO donors appeared to be effective in permanent and transient model of ischaemia, efficacy was limited to very early treatment within one hour of stroke induction [35].

**Fig. 4:**
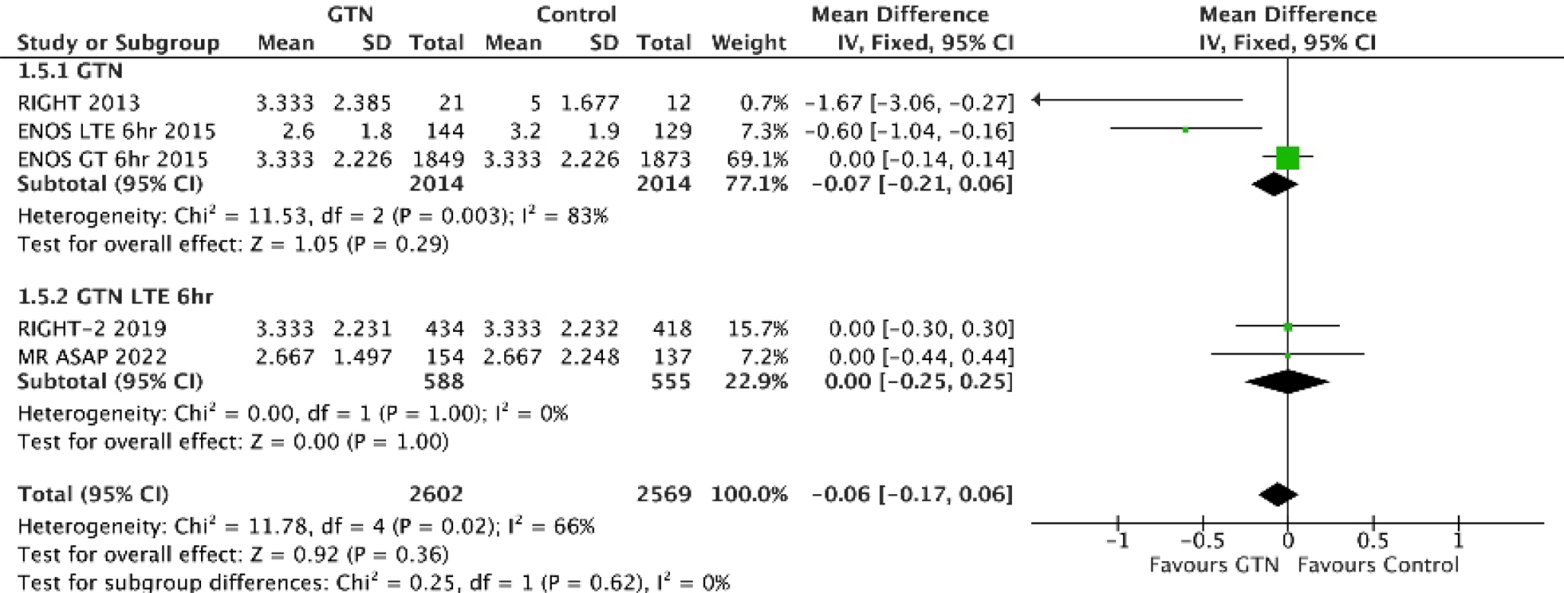
Effect of glyceryl trinitrate (GTN) on modified Rankin scale (low scores indicate recovery) at 90 days in acute mixed stroke. Data were extracted from the ENOS, RIGHT-1/2 and MR ASAP publications; [40–42, 52] where means and standard deviations were not available, they were estimated from medians and interquartile ranges using the method of Wan et al. using their published Excel calculator. [97] The mean difference (fixed effect) was calculated in Cochrane REVMAN software; a mean difference < 0 implies a good outcome. The overall effect of GTN in ENOS was neutral. [41] The *pre hoc* interaction test between early (< = /LTE 6 h) and later (> /GT 6 h) was significant. [41] The component of interest (< = 6 h) in ENOS was significant [44] as was the earlier phase-2 RIGHT trial. [40] The RIGHT-2 and MR ASAP follow-up trials were neutral. [42, 52]

GTN has been tested in six acute stroke trials [37–42]. The rationale for performing the rapid intervention with glyceryl trinitrate in hypertensive stroke trial-2 (RIGHT-2) ambulance-based pre-hospital trial was based on: i) plausibility related to the key vasoregulatory effect of NO; ii) observation that NO levels are lower in stroke and so replacement might be beneficial [43]; iii) positive evidence in a meta-analysis of preclinical studies of NO donors [35]; iv) a statistical interaction between the pre-defined subgroup of time from onset to randomisation and treatment (*p = *0.031) in the large Efficacy of Nitric Oxide in Stroke (ENOS) trial [41]; v) a positive result in those patients randomised within six hours of stroke onset in ENOS (Table 1) [44]; vi) a positive phase IIa ambulance trial of GTN [40]; and vii) a meta-analysis of the first five GTN trials showing benefit with treatment within 6 h of stroke onset [45]. Supporting these drivers was a trial of DCLHb, a NO scavenger which reduces vascular NO and increases endothelin-1 levels [46]; this trial was significant in a direction opposite of the hypothesized effect (not neutral) with associated worse mRS, Barthel and NIHSS [47]. Taken together, these results supported doing a large pre-hospital phase III trial of GTN. However, the subsequent Rapid Intervention with Glyceryl trinitrate in Hypertensive stroke Trial-2 (RIGHT-2) trial was neutral in both the target population of stroke and TIA and across the whole population that included mimics (Table 1, Fig. 4) [42]. The trial was negative in the ICH subgroup [48]; tended to be negative in the sub-population of IS/TIA, especially in those treated within 1 h; [49] was neutral in TIA [50]; and, surprisingly, positive in the mimic population [51]. Subsequently, a separate and independent trial, Multicentre Randomised trial of Acute Stroke treatment in the Ambulance with a nitroglycerin Patch (MR ASAP), was also neutral for this ultra-acute population of patients recruited prior to hospital admission and treated with GTN, albeit the study was stopped early for safety on recommendation by the data & safety monitoring board with only 325 patients enrolled [52]. The main weakness here is the 15 subgroups that were tested for interactions in the original ENOS trial [44] and so there is approximately a 54% chance (1 – (1 – 0.05) [15] = 0.54) possibility that at least one would be positive just by chance. A further follow-on trial, efficacy of nitric oxide-2 (ENOS-2), is investigating the feasibility of delivering GTN vs sham in patients with stroke presenting between 3 and 5 h of onset.

#### Nerinetide

Nerinetide (NA-1), a eicosapeptide that perturbs post-synaptic density protein 95 (PSD-95) and so affects synaptic scaffolding, inhibits neuronal excitotoxicity and reduces lesion damage in cultured neurones and mammals including primates [53]. Unfortunately, a formal meta-analysis of preclinical studies has not been published so it is unclear how many studies of NA-1 have been performed, their quality and whether publication bias is present and, so, whether subsequent clinical studies were warranted.

Nerinetide was safe in a phase II trial involving participants requiring endovascular treatment of intracranial aneurysms [54]. In the Endovascular Treatment for Small Core and Anterior Circulation Proximal Occlusion with Emphasis on Minimizing CT to Recanalization Times (ESCAPE-NA1) trial, 1105 patients were randomised with acute ischaemic stroke due to large vessel within 12 h (Table 1). The trial was neutral but a significant interaction was present between participants given alteplase versus those not given alteplase; specifically, participants receiving alteplase did not respond (mRS 0–2: aRR 0.97, 95% CI 0.87–1.08) whilst those not having alteplase appeared to respond positively (good outcome, mRS 0–2: nerinetide 59.3% vs placebo 49.8%, aRR 1.18, 95% CI 1.01–1.38). Although pre-specified, the interaction between treatment and alteplase was unexpected [55] and was not present in preclinical studies. In a subset of participants, nerinetide concentrations were lower following alteplase-treatment possibly reflecting plasmin degradation of nerinetide.

To further test nerinetide in hospitalised patients, the ESCAPE-NEXT phase III trial assessed whether nerinetide was beneficial in patients selected for endovascular revascularisation without thrombolysis (NCT04462536). The trial was presented at the World Stroke Conference 2023 (https://www.medscape.com/viewarticle/997312?form=fpf) suggesting that the prior observation in ESCAPE-NA1 regarding the interaction with alteplase was not relevant. This result does not necessarily signify the end of NA-1 since a separate phase IIc trial testing nerinetide in the pre-hospital environment (FRONTIER, NCT02315443) was also presented at World Stroke Conference 2023 and appeared to be positive with nerinetide benefitting functional outcome, especially in patients receiving reperfusion therapy (see above hyperlink). We now await peer-reviewed publication of these trials to better understand the findings and justify whether further trials are required. Since nerinetide can block nitric oxide signalling [56], it is noteworthy that ultraacute-prehospital treatment might improve outcome whereas exogenous administration of GTN/NO in the ambulance worsened outcome in ICH and IS [42, 48, 57]. Interestingly, successor plasmin-resistant PSD-95 inhibitors such as NN42 are immune to any interaction with thrombolytics [58] and are now being tested (https://neuronewsinternational.com/nono-announces-first-patient-dosed-with-nono-42-in-phase-1-study/).

#### Planned/ongoing trials

None of the exemplar follow-on trials described above were positive and we are not aware of any positive exemplar parings of trials. The recent positive ENRICH phase-2c trial of surgery for superficial ICH [34] might be considered to break this assertion; however, it was not a direct follow-on to the neutral STICH-1/2 trials and was based on a specific device rather than a number of different surgical approaches. In spite of this, we are aware of existing or planned trials chasing a positive subgroup component from a previous trial and three exemplars are presented here.

##### DM199

DM199 is a recombinant version of human tissue kallikrein (rKLK1), an endogenous serine protease that generates bradykinin and kallidin and so promotes vasodilation [59, 60]. A few studies have demonstrated that intravenous KLK1 improved outcomes after experimental strokes up to 24 h after stroke induction (reviewed in [59]) although there is no systematic review/meta-analysis of these to assess quality and publication bias. However, there is much existing clinical trial data based on human urinary kallidinogenase, i.e. KLK1 extracted from urine (uKLK1), which is approved in China for use up to 48 h after ischaemic stroke. uKLK1 reduced neurological impairment and improved long term outcome after acute ischaemic stroke in a meta-analysis of 24 clinical trials involving 2433 participants. [61].

In the small 92-patient ReMEDY-1 trial, DM199 was safe but did not alter functional outcome, as presented at the International Stroke Conference, 2021 (https://www.medpagetoday.com/meetingcoverage/isc/91701). [62] rKLK was associated with reduced stroke-in-evolution and, in a post hoc subgroup analysis, a tendency to improve functional outcome in participants who did not receive mechanical thrombectomy. As a result, the ongoing follow-on and larger ReMEDY-2 trial will recruit participants with IS who have not received thrombectomy with the primary outcome of excellent functional outcome (mRS < 2) (https://clinicaltrials.gov/study/NCT05065216, downloaded 17 November 2024).

### Uric acid

Uric acid is an endogenous antioxidant and low levels at the time of acute ischaemic stroke are associated with a poor outcome [63]. Hence, supplementation might benefit outcome. Uric acid has been reported to be neuroprotective in rats with transient focal ischaemia with reduced infarct size and improved blood brain barrier integrity in a systematic review [64]. However, this meta-analysis included many low quality studies and found significant publication bias [64]. Some preclinical studies suggested that coadministration or uric acid with alteplase was synergistic.

URICO-ICTUS was a phase 2b/3 trial of uric acid in patient treated with alteplase for acute ischaemic stroke [65]. Although the trial was neutral overall, uric acid improved functional outcome in a post hoc subgroup comprising 45 patients treated with mechanical thrombectomy [66]. A follow-up trial in the USA is planned in patients having mechanical thrombectomy (A Chamorro, personal communication). The rationale for this follow-on study is enhanced by the recently reported Stroke Preclinical Assessment Network (SPAN) trial [67]. This found that uric acid was the only intervention to survive a six-arm four-stage platform design in mice and rats and show efficacy with a positive primary outcome assessed as the corner test. Stroke was induced using insertion and then removal of a filament which mirrors thrombotic occlusion and then removal with thrombectomy; this fits with the thrombectomy subgroup reported for URICO-ICTUS [66]. A key spin-off of any future clinical trial of uric acid, if positive, would be initial validation of the SPAN preclinical platform model of developing new interventions for acute stroke.

### Glibenclamide

Blockade of the inducible sulfonylurea receptor-1-regulated calcium-ATP channel in endothelium, astrocytes and neurones with glibenclamide [68] reduces cerebral oedema in animal models of ICH [69] and similar effects have been seen in experimental stroke. Unfortunately, a metaanalysis of preclinical studies of glibenclamide for ICH found evidence of missing negative studies and the existing studies were of low quality and ad a high risk of bias [69]. Hence, clinical studies are based on potentially weak preclinical data.

A single-arm phase-2a study and phase-2b trial (GAMES-RP) showed feasibility and safety with intravenous glibenclamide in patients with large anterior circulation hemispheric infarcts who were at risk of developing cerebral oedema [70, 71] although the latter found no signal of efficacy for achieving a mRS of 0–4 without the need for decompressive hemicraniectomy. Recently the results of the international CHARM phase 2c trial of intravenous glibenclamide were presented at the European Stroke Organisation Conference 2024 (https://www.vjneurology.com/video/oocimi4lwgc-phase-iii-charm-trial-of-glibenclamide-for-large-hemispheric-infarction/). The trial was stopped early for commercial business reasons having recruited 431 patients of the intended 768. Although the trial was neutral for effects on mRS and death, glibenclamide had beneficial effects on mRS in prespecified subgroups of patients: those who received thrombectomy or thrombolysis and those with a medium-large stroke volume. A follow-up trial has been mooted involving patient groups who appeared to benefit in CHARM.

## Discussion

There are key learning points from these exemplars and a number of ‘rules’ can be formulated when deciding on whether to further test a positive subgroup. Equally, these can be considered as questions to ask of any trial with subgroup analyses [72].

1. Clinical trials should be based on sufficient numbers of heterogeneous preclinical studies and their data must be high quality and show no evidence of publication bias.
2. The subgroup should be plausible biologically.
3. The subgroup should be defined before analysis (*pre hoc*) [2].
4. The number of subgroups being tested should be limited to prevent false positive/type I error findings; or alternatively, adjustments (such as Bonferroni adjustments) should be made to protect from spurious findings arising from multiple testing.
5. The subgroup’s interaction test should be statistically significant.
6. The primary outcome in the subgroup’s component of interest should be statistically significant and further information published separately.
7. There is an explanation why the non-positive component(s) of a binary subgroup does not respond and may even suffer with treatment.
8. Other outcomes in the subgroup’s component of interest should show potential benefit, either showing statistical significance or at least a strong tendency.

Further ‘rules’ are highlighted elsewhere. [72–75].

### Preclinical data

This review has not examined preclinical evidence in depth but it is vital that clinical studies are built on high quality laboratory research which has then been meta-analysed and not found to show significant publication bias with apparent suppression of negative or neutral studies. However, as reviewed here and elsewhere [76], the presence of poor quality preclinical studies and significant publication bias that over-estimates preclinical efficacy in meta-analyses seriously damages the value of subsequent clinical studies. In addition, that multiple interventions are considered in early-phase trials with only those interventions showing potential efficacy proceeding to later trials implies the results of those moving forward are likely biased. For example, suppose that 100 compounds are considered in early-phase trials, and only 2 show results supporting later-phase trials. There are 2 reasons why these 2 trials could be showing potential efficacy: 1) the intervention truly works, and 2) out of the 100 interventions considered, by chance alone the estimated efficacy for these 2 interventions was estimated to be more beneficial than its true effect. The difference between the true effect and the falsely-estimated larger effect is a product of the selection of only “promising” interventions to move forward. As such, the strength of preclinical findings in “promising” interventions should be interpreted with caution. This is particularly relevant when the first sizable clinical trial is neutral and yet exhibits a positive subgroup interaction and component. It is much more likely that the neutral trial simply reflects that the intervention does not work and the research pipeline was misled by biased preclinical studies.

### Biological plausibility

It is vital that a positive subgroup component makes intellectual sense and is based on a rational indication [72–75]. Examples of biological plausibility follow here. First, earlier treatment is usually more effective than later treatment, as seen definitively for carotid endarterectomy, thrombolysis and thrombectomy [11, 15, 77]. Hence, a positive subgroup based on time from onset-to randomisation/treatment is attractive to test in a follow-on trial and this was justification for two exemplars presented here (piracetam and GTN). Second, replacement of a missing or reduced key endogenous factor offers plausibility, a justification that applies to uric acid and GTN. This contrasts with administration of magnesium, an endogenous modulator of the N-methyl-D-aspartate (NMDA) receptor complex, in IMAGES and FAST-MAG [78, 79] where blood levels are not usually reduced in acute stroke. Third, the benefit of thrombolysis and thrombectomy mean that there is less improvement left for another intervention to act on and it may be attractive to exclude such patients. This is one underpinning driver the for the ReMEDy2 trial of DM199. Fourth, interactions between alteplase, a thrombolytic, and the intervention being tested may either limit the effect of alteplase, as potentially seen with an IL-1 receptor antagonist [80], or limit the effect of the drug under test, as potentially seen for nerinetide in ESCAPE-NA1 [55]. Last, interventions might only work in certain types of IS; just as MT is only relevant for large vessel occlusion secondary to cardioembolism or large artery disease, it is possible that some interventions might only be effective in cerebral small vessel disease (cSVD). This is a possible explanation for the positive post hoc subgroup seen in ReMEDY-I and was explicitly tested in the lacunar intervention-2 (LACI-2) cSVD prophylaxis trial [81].

However, it is important to consider that both components in a subgroup with a positive interaction test may be plausible. For example, the Asymptomatic Carotid Artery Stenosis (ACAS) trial of carotid endarterectomy for asymptomatic carotid artery stenosis suggested that there might be a sex difference in the response to surgery with men possibly benefitting more than women (interaction *p = *0.10) [82], perhaps because women have smaller arteries than men so increasing the risk of surgery. But what if the opposite finding had been true? It might have been explained that men have more advanced atherosclerosis and surgery in men is therefore more dangerous. Hence, it is important that plausibility is both explained and the expected direction of difference is defined *a prior*.

### Choice of subgroups

It is vital that potential subgroups of interest are identified in the statistical analysis plan prior to data lock and analysis, so-called confirmatory subgroup analyses [3, 10, 75, 83]. Further, the direction of effect should be pre-specified [75]. Subgroup findings based on post hoc analyses (exploratory subgroup analyses [10]) are notoriously suspect and most trials could find a ‘sensible’ post hoc subgroup interaction of potential interest with sufficient data-dredging. If post hoc subgroups are assessed then this should be clearly identified. [3]

Choosing subgroups and how many to include during the design stage of a trial is challenging and yet may not attract much time or attention. If stratification and/or minimisation [84] is used during randomisation, then the subgroups should include the same variables, typically including age, sex, severity and time to randomisation. There may be other variables of clinical relevance that should be included, e.g. use of reperfusion therapy [55] or prior nitrate exposure in a trial of a NO donor [44]. Vitally, all pre-specified subgroups must have their interaction test results reported [75].

### Number of subgroups

Accepting that subgroups should be chosen on the grounds of plausibility, stratification and minimisation, and prognostication, having too many risks that one or more interaction tests will be significant purely due to chance [10], as might have happened in ENOS which had 15 subgroup interaction tests resulting in more than a 50% chance that at least one would be statistically significant [44]. Although there is little consensus on how many subgroups is acceptable in a large trial, a minimalistic and perhaps extreme rule of thumb is that subgroup analyses should be limited to one or two purely because the positive predictive power of any analysis falls dramatically as more are performed [83]. Alternatively, the interaction test can be considered positive taking account of the number of subgroups following a Bonferroni procedure: one subgroup interaction would be assessed at *p < *0.05, two at *p < *0.025 and ten at *p < *0.005. These approaches, and other more sophisticated ones [10], all aim to control for the type I error rate.

### Number of components in a subgroup

Most subgroups comprise two components, e.g., male vs female, or early vs late treatment. In this scenario, when a significant statistical interaction is present, it is vital to be able to consider why the intervention might have caused harm in the component associated with the poor outcome as well why it might have caused benefit in the other component. In this respect, two component subgroups are especially prone to chance as well as interpretation bias. By example, in the CLASS trial of clomethiazole, the subgroup with a significant interaction term had benefit in the total anterior circulation syndrome (TACS) group, OR 0.62 (0.43, 0.88) *p = *0.008, *n = *545, and a tendency to hazard in the non-TACS group, OR 1.15 (0.85, 1.54) *p = *0.36, *n = *808; the obvious question is why clomethiazole might be potentially hazardous in non-TACS patients and yet potentially beneficial in TACS? Without answers to both questions, it is questionable whether any follow-on trial should have been performed. In contrast, significant subgroup interactions based on three or more components, especially where there is a gradient, are less likely to be based on chance.

An obvious drawback of creating more strata for a factor is there will be a smaller average sample size in each of these strata, with an associated decreased level of precision of the estimates in each strata. In addition, having a larger number of strata increases the degrees-of-freedom for the assessment of interaction, making it more difficult to detect true effect modification (i.e., more likely to have a type-II error in the assessment of interaction).

However, a gradient towards benefit or hazard across three or more ordered components is much more persuasive that the subgroup observation might be real. For example in ENOS, treatment within 6 h was associated with benefit, OR 0.51 (95% CI 0.32–0.80) [85], with neutral effects seen in all the other time components (6–12, 12–24, 24–36 and 36–48 h) [41]. The absence of a gradient across multiple components would, of course, suggest the interaction was related to chance. And testing heterogeneity in subgroups has much lower power than the whole trial and so is inevitably underpowered. If the subgroup is divided into three or more components then the statistical power for each will fall and so significant findings may be missed. For example, the < 6 h component of the time to randomisation subgroup in ENOS was one-of-five components and only comprised 6.8% of the sample size (Table 1) [41]. In this particular example, the fact that treatment showed benefit in this small hyperacute component [44] made it particularly interesting.

### Interaction testing and level of significance

It is vital that subgroup analyses are based on formal tests of interaction [3] and that the interaction test for the subgroup of interest is statistically significant at the pre-determined assessment level determined a priori. Historically, interaction tests used *p < *0.05 as a cut-off but there is a trend to using *p < *0.10 on the grounds that the "cost" of making a false negative/type II error is potentially greater than a false positive/type I error, especially since false negative interaction tests may occur due to low statistical power secondary to a low sample size [83].

In contrast, *p < *0.05 may even be too large as reported in a European phase II/III trial of trafermin (basic fibroblast growth factor); this reported a positive post hoc time-based interaction test, suggested that multiplicity of testing should be accounted for with more stringent testing [86]. This has also been suggested in the context of surgical trials [72]. Although it may still be appropriate to test for interactions at *p < *0.05, testing across a large number of subgroups will lead to an increased risk of false positive findings. In summary, the number of groups should be limited, interaction testing should be performed at *p < *0.05 or *p < *0.10 and the potential number of false positive results based on the number of subgroups tested reported [87].

### Analysis of the subgroup component of interest

It is vital that analysis of the ‘positive’ subgroup component is statistically significant with appropriate reporting of the odds ratio/hazard ratio and 95% confidence intervals. Ideally, a secondary publication will then provide detailed information on the subgroup overall and specifically the component of interest. Further, effects of the intervention on secondary outcomes should also be reported; if these also show benefit, such internal consistency will add strength to the argument to perform a second trial. Such secondary publications were done for clomethiazole and GTN [30, 44].

We must also keep in mind that when there are striking effects in one subgroup component, this may be counterbalanced by detrimental effects in other subgroup components which may be as clinically important as the positive subgroup result. Nevertheless, the unexpected finding of an interaction is an important finding that needs to be reported for the purpose of hypothesis generation, even if the interaction just fails statistical significance.

### Effects in secondary outcomes

It is to be expected that analyses of a subgroup component should show beneficial effects in outcomes other than just the primary. Depending on what the primary outcome was, examples might be positive effects on one or more of death, impairment, disability/activities of daily living (Barthel index), dependence (modified Rankin scale), quality of life, cognition and mood, as seen in Table 1.

### Sample size of the follow-on trial

A counter-intuitive observation is that follow-on trials may need to be larger than the first trial, especially if the positive finding in the first trial’s subgroup component has a significance near to *p = *0.05. If the follow-on trial is similarly sized, the likelihood that the power to confirm the first trials significant finding at *p = *0.05 is only 50%, i.e. there is a 50% chance that the effect will be smaller and a 50% chance that it will be larger. In this scenario, confirming the borderline significant finding requires a substantial increase in sample size relative to the size of the positive subgroup. In STICH, the apparent beneficial effect of surgery in patients with a superficial haematoma was almost significant with *p = *0.051 (Table 1). Hence, the follow-on STICH-2 trial should have been larger than STICH although, in reality, STICH-2 was smaller at *N = *601 than STICH at *N = *1033. This observation is important since the meta-analysis of the population of participants with superficial haematoma in STICH-1 and 2 is marginally significant (Fig. 3) and a larger follow-on trial might have given more robust and positive results.

### Publication of the follow-on trial

As with all completed trials, any follow-on trial must be published. Unfortunately the PASS-2 trial of piracetam was never published and so no learnings can be made from it and the data cannot contribute to meta-analysis [22]. Such publication bias is unethical and uninformative since patients have been exposed to potential hazard but without any benefit to patient care or the scientific literature.

### Subgroups derived from meta-analyses

This review has focussed on follow-on trials driven by the results of a positive subgroup interaction in a previous trial. The reservations expressed here with this approach apply less to trials based on pre-defined subgroups from meta-analyses of trials. Guidelines on the approach to performing and interpretating subgroup analyses within meta-analyses are given by the Cochrane Collaboration [88]. For example, adequately powered studies based on subgroup results from analyses of pooled trial data are more likely to be successful, as seen with a meta-analysis of the ATLANTIS, ECASS and NINDS trials of alteplase [89] which identified likely efficacy in the 3–4.5 h time window which then led onto the positive ECASS-III trial [90]. However, the meta-analysis of the first five GTN trials showing benefit with treatment within 6 h of stroke onset [45] did not lead to a positive prehospital trial [42].

## Conclusions

Although some of the exemplars given here followed many of the recommended scientific considerations when designing and interpretating subgroup analyses, the follow-up trials were still neutral which raises the question of why. The most likely reason is a chance finding in the original trial, i.e., the interaction test in the first trial was falsely positive. Explanations will vary but post hoc identification of subgroups or their components and having too many subgroups are likely explanations.

A second cause is that some of the biologically plausible reasons underlying trials may be wrong. Notably, the predictive value of preclinical data is poor since more than a thousand neuroprotectant animal models of stroke have been reported [91] but none have shown clinical efficacy and, indeed, many caused harm [7]. Further, the “earlier is better” paradigm for reperfusion interventions may not apply to other families of interventions. For example, although a meta-analysis based on individual patient data of all the GTN trials (including the ENOS early subgroup and RIGHT trial) supported earlier is better [45], RIGHT-2 found that ultra-acute treatment within 2 h was potentially hazardous in both ICH and IS [42, 57]. Although the reasons for this are unclear, GTN might inhibit the primary and secondary stages of haemostasis and so increase haematoma expansion in ICH [92], and reduce perfusion in the presence of lost auto-regulation in IS. Hence, an “early but not too early” paradigm may be appropriate for some interventions, as also reported for physical therapy [93].

In summary, prospective testing of a positive component in a subgroup in an earlier trial comes at high risk since it is usually unsuccessful. It is vital that the subgroup is biologically plausible, supported by properly designed and reported preclinical studies where relevant, based on a significant statistical interaction adjusted for multiple testing, accounting for negative as well as positive subgroup components and describes the positive subgroup component in detail. Notice that we are not saying that no second trial chasing a positive subgroup can ever be positive, indeed it is inevitable that chasing a positive subgroup will eventually lead to a positive follow-on trial. However, this may be a less frequent outcome and investigators should be aware of the risks.

## Data Availability

Not applicable – all quoted information has been published.

## Abbreviations

ACAS: Asymptomatic carotid artery stenosis
CI: Confidence intervals
CLASS: Clomethiazole acute stroke study
ESCAPE: Endovascular treatment for small core and anterior circulation proximal occlusion with emphasis on minimizing CT to recanalization times
ENOS: Efficacy of nitric oxide in stroke
ENRICH: Early minimally invasive removal of intracerebral haemorrhage
GABA: Gamma-aminobutyric acid
GTN: Glyceryl trinitrate
ICH: Intracerebral haemorrhage
IS: Ischaemic stroke
KLK: Kallikrein
MR ASAP: Multicentre randomised trial of acute stroke treatment in the ambulance with a nitroglycerin patch
mRS: Modified rankin scale
NA-1: Nerinetide
NMDA: N-methyl-D-aspartate
NO: Nitric oxide
OR: Odds ratio
PASS: Piracetam acute stroke study
PSD-95: Post-synaptic density protein 95
RIGHT: Rapid intervention with glyceryl trinitrate in hypertensive stroke trial
RR: Risk ratio
SPAN: Stroke preclinical assessment network
SSS: Scandinavian stroke scale
STICH: Surgical trial in intracerebral haemorrhage
TACS: Total anterior circulation syndrome
